# Seamless integration for enhanced seizure prediction using *HybridConvMobileNet* on Typhoon HIL

**DOI:** 10.1038/s41598-025-29393-5

**Published:** 2025-11-27

**Authors:** Prabhat Kumar Upadhyay, Priyaranjan Kumar, Manoj Kumar Panda, Aswini Kumar Samantaray

**Affiliations:** 1 Department of Electrical and Electronics Engineering, Birla Institute of Technology, Ranchi, Jharkhand 835215 India; 2 Department of Electronics and Communication Engineering, GIET University, Gunupur, Odisha 765022 India; 3 Department of Electronics and Communication Engineering, Manipal Institute of Technology Bengaluru, Manipal Academy of Higher Education, Manipal, India

**Keywords:** Convolutional Neural Networks (CNN), Epilepsy prediction, Frequency-domain features, Real-time seizure detection, MobileNet, Electrical and electronic engineering, Biomedical engineering

## Abstract

Real-time seizure prediction is essential for enabling timely interventions that significantly improve patient outcomes. Therefore, in this present work, we have introduced *HybridConvMobileNet*, a novel hybrid model that integrates 1D convolutional neural networks (CNN) with the MobileNet model to achieve efficient and accurate seizure prediction. The proposed model uses 1D Short-Time Fourier Transform (STFT) coefficients from pre-processed EEG data as input features. In the proposed algorithm, the developed 1D CNN framework captures the critical spatial features from frequency-domain EEG data, while MobileNet network enhances computational efficiency and speed, making the model highly appropriate for real-time applications. The efficacy of the developed model is corroborated on the Children’s Hospital Boston-Massachusetts Institute of Technology (CHB-MIT) and Siena benchmark datasets. On the CHB-MIT dataset, the model reached 99.70% accuracy, 99.31% sensitivity, and a 99.43% F1-score, while on the Siena dataset, it reached 99.67% accuracy, 99.08% sensitivity, and a 99.57% F1-score, outperforming eight existing methods across both datasets. Furthermore, real-time implementation on the Typhoon HIL emulator with embedded C2000 microcontrollers demonstrated a low mean detection latency of 0.1 to 1 second, underscoring its potential for clinical applications in seizure monitoring and control.

## Introduction

Real-time automated seizure detection and its hardware implementation is a multidisciplinary field that requires collaboration and expertise from biomedical researchers, electrical engineers, and neurologists. Addressing this challenge is crucial, as around 1% of the global population is impacted by epileptic seizures, with 20% to 40% of these patients becoming resistant to antiepileptic drugs^1^. Epilepsy is characterized by seizures, which are sudden, uncontrolled electrical disturbances in the brain. These disturbances can significantly impact higher brain functions, leading to profound but temporary changes in behavior, movement, sensation, and consciousness^2^. By applying metal electrodes to the scalp and recording an electroencephalogram (EEG), one can identify the excessive electrical activity in the brain that causes seizures^3^. Analyzing EEG signals is the primary method for detecting seizures. Epilepsy patients experience four distinct states: the ictal state during a seizure, the preictal state before a seizure, the post-ictal state following a seizure, and the inter-ictal state when no seizure activity is present^4,5^. The main objective of seizure predictions is to identify the pre-ictal state in advance^6^, so classifying pre-ictal and inter-ictal states is essential to seizure prediction. Unique EEG features, which vary from patient to patient, are essential for accurately distinguishing between these states. State-of-the-art seizure prediction techniques often involve sophisticated software methods, which can be broadly classified into time-domain analysis, frequency-domain analysis, and non-linear dynamics^7–9^.

Given the unpredictable nature of seizures, epilepsy has significant psychological and social impacts, and in severe cases, it can be life-threatening. Predicting epileptic seizures could dramatically enhance the quality of life for patients by providing early warnings, allowing sufficient time to take appropriate action, and paving the way for new treatment methods and strategies to better understand the disease. In this context, the seizure prediction problem can be framed as distinguishing between the pre-ictal and inter-ictal brain states. When the pre-ictal state is detected amid the typically dominant inter-ictal state, the indicator is triggered to signal the potential onset of a seizure. In contrast, the proposed *HybridConvMobileNet* model effectively addresses these limitations by utilizing 1D STFT in matrix form, significantly reducing computational complexity while preserving high predictive accuracy. Moreover, the developed model has been uniquely validated on the Typhoon HIL real-time emulator, ensuring optimized performance for hardware implementation and real-time seizure prediction.

### Contributions of this study

The main contributions of this work are summarized as follows:

1. A novel *HybridConvMobileNet* model is introduced, combining 1D-CNN and MobileNet architectures to achieve efficient and precise seizure prediction.
2. The 1D-CNN is designed to extract diverse features while leveraging the lightweight structure of the MobileNet framework, ensuring robustness and computational efficiency.
3. The model’s suitability for low-power, real-time clinical applications is demonstrated through deployment on the Typhoon HIL emulator with an embedded C2000 microcontroller.
4. High performance is achieved on both the CHB-MIT and Siena datasets, with low latency (0.1–1 s), underscoring the model’s clinical applicability.
5. The model’s generalization has been validated through cross-dataset evaluation, demonstrating robust performance on unseen data.

The article is structured as follows. The *Related Work* section reviews the literature on seizure prediction. The *Dataset and Methodology* section provides a detailed description of the developed algorithm and the dataset used. The *Experimental Results* section offers an empirical analysis, encompassing both the discussion and the ablation study outcomes. Finally, the Conclusion section summarizes the findings and suggests directions for future research.

## Related work

The literature identifies various methods for seizure prediction, highlighting advances in EEG feature extraction and modeling. Time-domain features, such as statistical moments and zero-crossing rates, effectively detect early seizure signs^10^. Frequency-domain features from STFT and time-frequency methods like CWT offer detailed analyses of EEG signals^11,12^. Nonlinear dynamics such as Higuchi’s fractal dimension and sample entropy capture seizures’ chaotic nature^13^. Brain connectivity measures and techniques like EMD enhance signal decomposition and detection accuracy^14–16^. Dimensionality reduction via PCA and ICA, along with ML algorithms like MLP, SVM, k-NN, and LDA, also contribute to improved classification^17–24^. However, the variability in EEG patterns across patients remains a significant challenge for required feature identification.

Deep learning has offered significant promise in seizure prediction, notably improving accuracy and early detection. Amrani et al.^25^ provided a hybrid DNN model, merging CNNs with LSTMs, that achieved notable accuracy on three EEG datasets CHB-MIT (92.8%), Siena Scalp (92.7%), and Helsinki Neonatal (86.4%). However, the model’s reliance on undersampling could cause data loss and its fixed EEG configurations may limit its scalability and risk overfitting.

Jana et al.^26^ introduced a 1D-CNN method paired with the NSGA-II algorithm for a lightweight, energy-efficient seizure prediction device. This approach significantly reduces EEG channels while maintaining high prediction accuracy (96.51%), though it requires considerable computational resources during training.

Wei et al.^27^ introduce the AFC-GCN, a compact Graph Convolutional Network that leverages adaptive functional connectivity for seizure prediction. This model effectively integrates spatial and temporal analyses, achieving high accuracy on the CHB-MIT (98.15%) and Siena datasets (96.80%), with sensitivities of 98.02% and 97.70% and low false positive rates of 0.0172 and 0.042 respectively. Traditional CNNs often struggle to utilize functional connectivity effectively, limiting their ability to detect detailed patterns.

Kapoor et al.^28^ introduced an IoT-based model that combines a Deep-CNN with a hybrid cuckoo finch optimization strategy for seizure prediction. The model achieved specificity of 92.52% accuracy of 97.76% and sensitivity of 95.63%. Despite its effectiveness a significant drawback is the model’s high computational demand during training.

Bell et al.^29^ introduced an approach using EEG for epileptic seizure detection focusing on developing an inter-intra Head-Body-Tail (HBT) feature extraction method combined with a Complementary Convolutional Neural Network (CP-CNN). This CP-CNN features two parallel traditional 1D-CNN sections with complementary convolutional kernels to preserve critical frequency components often lost in standard CNNs. The model achieved an accuracy of 98.17% and specificity of 98.25% on the CHB-MIT dataset and accuracy of 98.76% and specificity of 98.77% on the Siena dataset. However, its focus on high-frequency components may reduce effectiveness in scenarios needing low-frequency information for accurate seizure detection.

Sonawane et al.^30^ introduce a deep CNN and smart societal optimisation algorithm to enhance epileptic seizure prediction, achieving 86.73% accuracy on the CHB-MIT database and 87.67% accuracy on the Siena Scalp EEG Database.

Wei et al.^31^ suggested a Self-Supervised Graph Network with Time-Varying Functional Connectivity (SSGN-TVFC) that leverages wavelet-based temporal–spectral analysis, adaptive graph convolution, and contrastive learning. On the CHB-MIT dataset, this model achieved an AUC of 0.991, an accuracy of 0.990, a sensitivity of 0.992, and a very low FPR of 0.012, clearly highlighting the advances in pediatric scalp EEG seizure prediction. However, its reliance on complex graph construction and self-supervised pretraining leads to computational overhead, which restricts direct deployment in real-time embedded systems. Similarly, Chavan et al.^32^ proposed an optimized deep dual adaptive CNN-HMM classifier that combines wavelet decomposition, Human Learning Optimization (HLO) for electrode selection, and hybrid feature extraction techniques (TQWT, Hjorth, and statistical measures). Their approach achieved 99.46% accuracy on the CHB-MIT dataset and 94.53% on the Siena dataset, outperforming several conventional CNN and HMM methods. Nonetheless, this dual adaptive design introduces high computational cost, and importantly, patient-wise cross-validation was not conducted, limiting the evidence for robust generalization in clinical practice.

Cherian and Kanaga^33^ addressed hybrid deep learning models combining convolutional and attention-based mechanisms for EEG seizure detection. They introduced three architectures: CNN-BiGRU, CNN-BiLSTM, and a convolutional self-attention model, evaluated on the CHB-MIT dataset. The convolutional self-attention model achieved 99.04% accuracy, 96.06% sensitivity, and 99.11% specificity, outperforming conventional CNN and RNN approaches. The study is limited to a single dataset without patient-wise cross-validation, raising concerns about generalizability to clinical settings. The related works discussed face challenges such as high computational demands during training, reliance on under-sampling that can lead to data loss and fixed EEG configurations that limit generalisation. Many machine learning models also struggle with scalability and real-time efficiency especially with computationally intensive feature extraction methods like STFT and (continuous wavelete transform) CWT, underscoring the need for more efficient, scalable solutions.

The motivations of the proposed model are highlighted as follows:

1. Existing models have high computational demands which limit their real-time applicability necessitating a more efficient approach.
2. Models often experience data loss and poor generalization due to fixed EEG configurations calling for a more robust solution.

In this work, we have developed a unique model where using 1D STFT in matrix form simplifies computations while maintaining high prediction accuracy, addressing inefficiencies in traditional methods. The proposed model is optimized for real-time hardware implementation on the Typhoon HIL C2000, offering a solution for seizure detection systems.

## Developed technique

**Fig. 1:**
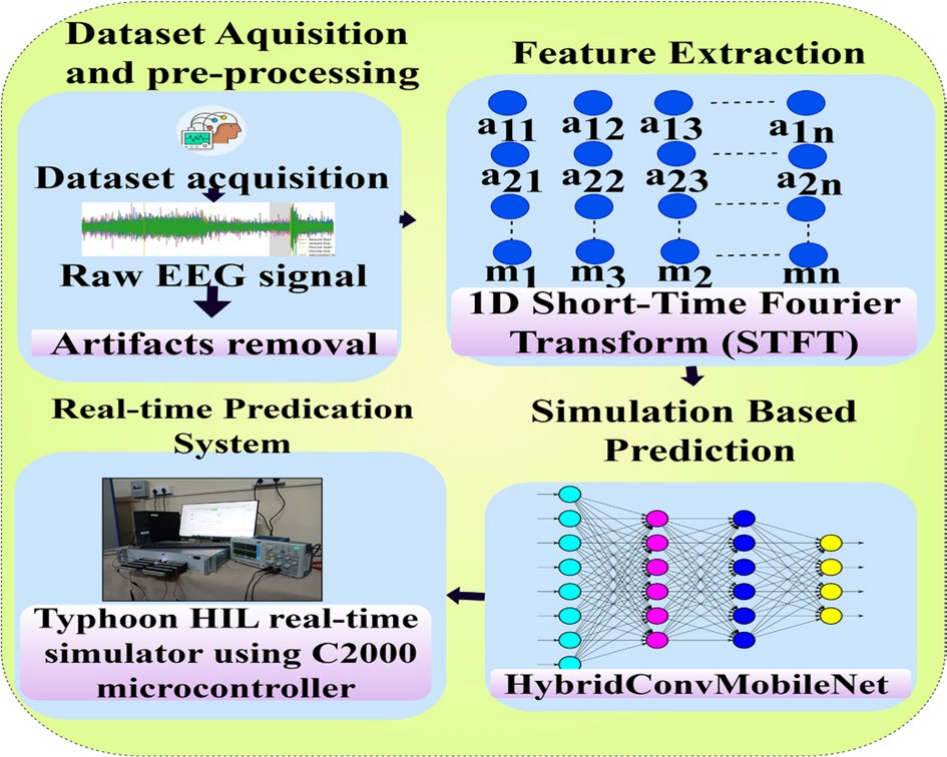
Illustration of the flow chart of this article.

This article presents a novel method for epileptic seizure prediction, structured into four key stages: dataset description and preprocessing, feature extraction, the proposed model, and real-time handling of ictal events along with an early warning strategy. Figure 1 illustrates the overall structure of the proposed method, and the subsequent sections provide a detailed explanation of each stage.

### Dataset details

#### CHB-MIT dataset

The CHB-MIT EEG dataset, publicly accessible through the PhysioNet repository (https://physionet.org/content/chbmit/1.0.0/), includes 844 hours of continuous EEG recordings from 23 pediatric patients aged 1.5 to 19 years with approximately 200 seizures captured using a universal bipolar montage of 24–27 EEG channels while on anti-seizure drugs and sampled at 256 Hz.

#### Siena scalp EEG dataset

The Unit of Neurology and Neurophysiology at the University of Siena, Italy, collected the openly accessible Siena database, also available on PhysioNet (https://physionet.org/content/siena-scalp-eeg/1.0.0/), which focuses on seizure prediction research. This dataset features scalp EEG recordings from 14 adult participants recorded shortly after they stopped antiepileptic medication, with most recordings continuous and captured using 29 channels at a 512 Hz sampling rate. Data from 10 participants have been used in this study.

### Dataset preparation and preprocessing

In this study, the preictal phase has been defined as EEG data from 35 to 5 minutes preceding a seizure, deliberately excluding the 5-minute intervention period to ensure a clean separation from ictal activity. The interictal phase has been defined as EEG segments occurring at least 4 hours before or after a seizure event, following established seizure prediction guidelines^27^. To preserve label integrity, transitional regions not clearly belonging to either class have been intentionally excluded. Specifically, the period between 4 hours and 35 minutes before a seizure has been treated as a buffer zone without any assigned label. Similarly, the final 5 minutes prior to seizure onset have been excluded to avoid contamination with rapidly evolving seizure dynamics. During real-time streaming, these segments pass through the system without active classification, and a prediction lockout mechanism has been employed to suppress redundant alerts during these intervals. This labeling strategy enhances robust training and minimizes misclassification near critical transitions.

Given the inherent class imbalance with interictal data dominating, several balancing techniques have been employed. EEG recordings have been preprocessed using a 0.1–80 Hz band-pass filter combined with a 50 Hz notch filter to remove baseline drift, muscle artifacts, and power-line interference. All available EEG channels have been included in the analysis to capture the full spatial distribution of seizure-related dynamics. To reduce variability in amplitude across sessions and patients, all EEG segments have been normalized on a patient-wise basis using z-score normalization:

1 $$\begin{aligned} z = \frac{x - \mu }{\sigma } \end{aligned}$$

where *x* is the raw EEG value, $\mu$ and $\sigma$ are the mean and the channel standard deviation. This procedure standardizes the EEG data to zero mean and unit variance, ensuring stable model training and preventing bias due to patient-specific amplitude differences. The transformation has been applied consistently across all patients in the CHB-MIT and Siena datasets. Synthetic preictal samples have been generated through the Synthetic Minority Over-sampling Technique (SMOTE)^34^, producing new samples via linear interpolation:

2 $$\begin{aligned} x_{\text {new}} = x_i + \lambda (x_j - x_i), \quad \lambda \in [0,1] \end{aligned}$$

where $x_i$ and $x_j$ are minority class samples and $\lambda$ is a random scalar.

Further preictal augmentation has been achieved using a Sliding Window technique with 80% overlap:

3 $$\begin{aligned} N_w = \frac{T - o}{w - o} \end{aligned}$$

where $N_w$ denotes the number of generated windows, $T$ the total segment length, $w$ the window size, and $o$ the overlap length.

To balance the dataset, Random Under-sampling has been applied to interictal samples according to:

4 $$\begin{aligned} N_{\text {interictal}}^{\text {new}} = \min (N_{\text {interictal}}, N_{\text {preictal}}) \end{aligned}$$

ensuring that the number of interictal samples matches the preictal samples post-augmentation.

By combining SMOTE, the Sliding Window technique, and Random Under-sampling, a balanced and clean dataset has been prepared, promoting robust seizure prediction and real-time applicability without bias from ambiguous temporal regions.

### Feature extraction

Standard EEG studies often employ 2D STFT spectrograms that map frequency content across both time and frequency axes, but such representations are computationally heavy and storage-intensive. In this work, STFT coefficients are instead arranged in a 1D matrix form that maintains temporal ordering while encoding frequency resolution along one axis. This compact representation reduces redundancy, avoids the need for 2D convolutional pipelines, and directly integrates with 1D CNN filters. By preserving temporal fidelity while lowering dimensionality, the 1D matrix form ensures efficient processing suitable for real-time deployment on Typhoon HIL C2000.

In this work, a 1D Short-Time Fourier Transform (STFT)^35^ extracts frequency-domain features from EEG signals utilizing a 2-second window with a 1-second overlap to capture time-varying frequency components in the 0.1–80 Hz range for seizure detection. The 1D STFT converts a time-domain signal into its time-frequency representation and can be represented as

5 $$\begin{aligned} X(t,f) = \int _{-\infty }^{\infty } x(\tau ) \omega (t-\tau ) e^{-j2\pi f \tau } d\tau \end{aligned}$$

In this approach $X(t,f)$ stands for the time-frequency view of the signal where $\omega (t)$ is the window function is $f$ the frequency and $\tau$ the time shift. This process generates a 1D STFT matrix that provides a compact efficient snapshot of the signal’s spectral details feeding into the model to enhance computational efficiency and preserve high-resolution information vital for accurate seizure classification.

### Proposed *HybridConvMobileNet* model

The *HybridConvMobileNet* is a unique framework designed to efficiently and accurately predict seizures using EEG signals. It combines the feature-capturing capabilities of Convolutional Neural Networks (CNNs) with the lightweight structure of MobileNet^36^. This integration maximizes efficiency, reduces processing demands, and enhances suitability for real-time applications. CNN layers extract the rich spatial properties of EEG inputs while MobileNet levels optimize the model’s performance. By utilizing 1D STFT features as input, the *HybridConvMobileNet* captures essential time-frequency information for precise epileptic seizure prediction.

**Fig. 2:**
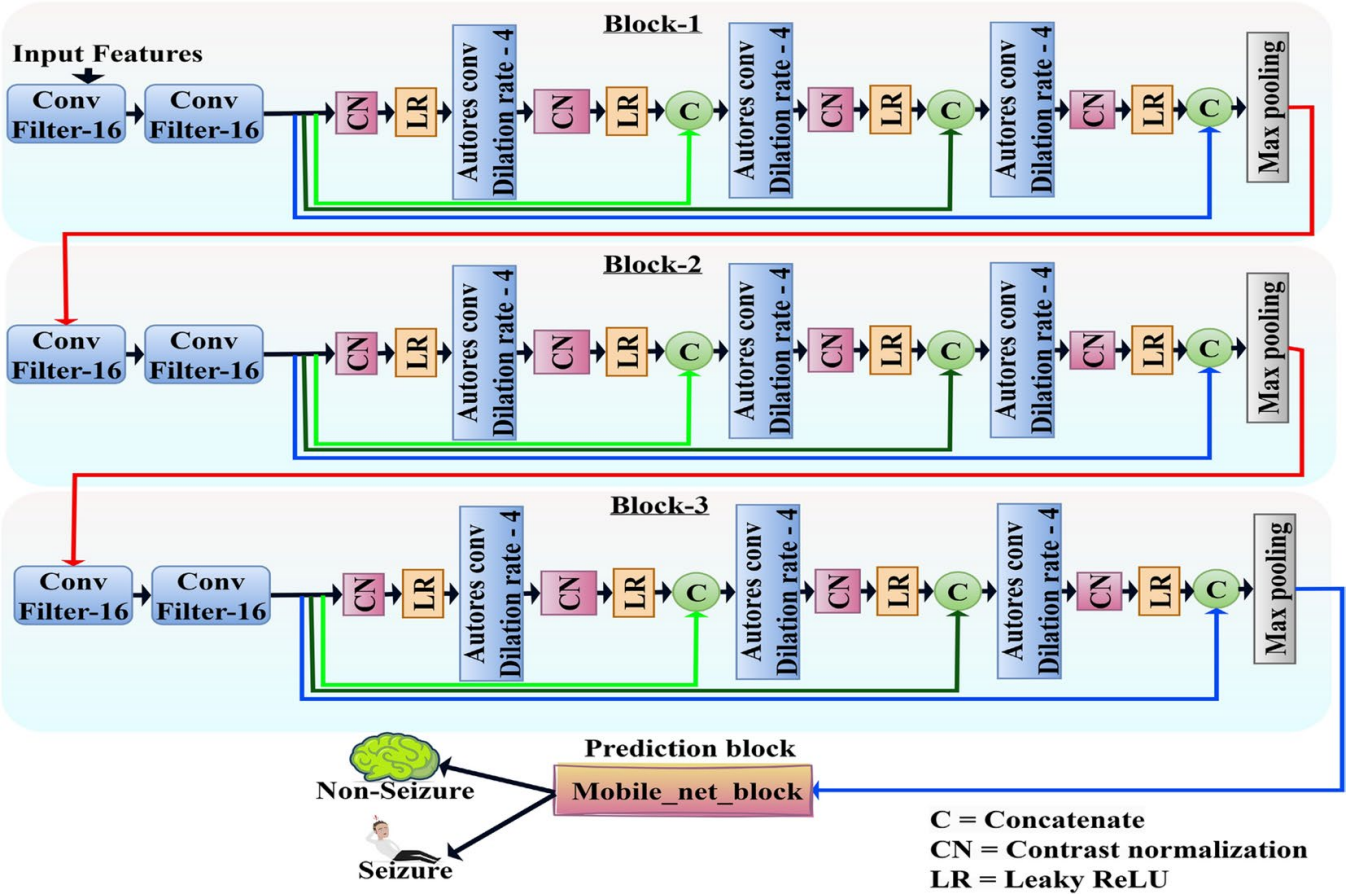
Schematic diagram of the *HybridConvMobileNet* architecture.

Figure 2 illustrate the visual representation of the propsed *HybridConvMobileNet* architecture highlighting its integration of convolutional blocks and MobileNet. Each component and its role within the model will be detailed in subsequent sections of this article.

#### Convolution layer

In the *HybridConvMobileNet* model, the architecture of convolutional layers is tailored to process 1D STFT inputs derived from EEG signals. Filters of varying sizes across these layers are strategically configured to enhance feature extraction for efficient real-time seizure detection. Block 1 uses 16 filters with increasing dilation rates to capture basic temporal patterns without increasing computational demands. Blocks 2 and 3 escalate the number of filters to 32 and 64 respectively.

6 $$\begin{aligned} y[k,t] = \sum _{s=0}^{S-1} f[t+s] \cdot W_k[s] + b_k \end{aligned}$$

here $y[k,t]$ is the output from the k-th filter at time $t$, $f[t]$ is the STFT-transformed input, $W_k[s]$ is the kernel for the k-th filter, and $b_k$ is the bias.

This formulation is crucial as it shows how the network processes and interprets complex spatial patterns in EEG data.

#### Contrast normalization

This process typically involves adjusting the input data to achieve more uniform statistics, often by reducing local variations in signal amplitudes, particularly in EEG signal processing. The contrast normalization can be expressed as

7 $$\begin{aligned} x' = \frac{x - \mu }{\max (\sigma , \epsilon )} \end{aligned}$$

where, $x$ is the original signal, $\mu$ represents the mean, $\sigma$ is the standard deviation, $\epsilon$ is a small constant to prevent division by zero, and $x'$ denotes the normalized output.

#### LeakyReLU

Leaky ReLU enhances the traditional ReLU activation function by allowing a small non-zero gradient when the unit is inactive and the input is less than zero. This is useful in preventing the ‘dying neurons problem’ in neural networks where neurons permanently output zeros due to negative inputs. The Leaky ReLU activation function is described as

8 $$\begin{aligned} f(x) = {\left\{ \begin{array}{ll} x & \text {if } x> 0 \\ \alpha x & \text {if } x \le 0 \end{array}\right. } \end{aligned}$$

where, $\alpha$ is a small coefficient typically set to 0.01 that ensures a slight gradient for negative input values. This allows continuous network learning even with negative inputs preventing neurons from ceasing to activate and adapt during training.

#### Dilated convolution layer

The dilated convolution layer, also known as atrous convolution, broadens the model’s ability to integrate wider contextual information from EEG data. Increasing dilation rates in sequential layers enable capturing extensive temporal patterns associated with seizures while preserving high resolution. The dilated convolution is expressed as

9 $$\begin{aligned} y(t) = \sum _{s=-a}^{a} x(t + r \cdot s) \cdot w(s) \end{aligned}$$

Here, $y(t)$ is the dilated convolution layer’s output, $x(t)$ is the input for dilated convolution layer, $w(s)$ the filter weights, $r$ the dilation rate, and $s$ the extent of the filter adjusted by dilation. This structure allows for deeper insight into EEG signal patterns relevant to seizure activity.

#### Concatenation layer

The Concatenation layer in the *HybridConvMobileNet* model is essential for integrating features from different convolutional layers by merging multiple feature maps into a single larger map along the channel dimension.

10 $$\begin{aligned} Y = \text {concat}(X_1, X_2, \dots , X_n) \end{aligned}$$

where $X_1, X_2, \dots , X_n$ are the individual feature maps from earlier layers, and $Y$ is the concatenated output. This layer enhances the model’s depth and analytical accuracy by preserving a broad spectrum of features, ensuring efficient information flow across the network.

#### MobileNet block

**Fig. 3:**
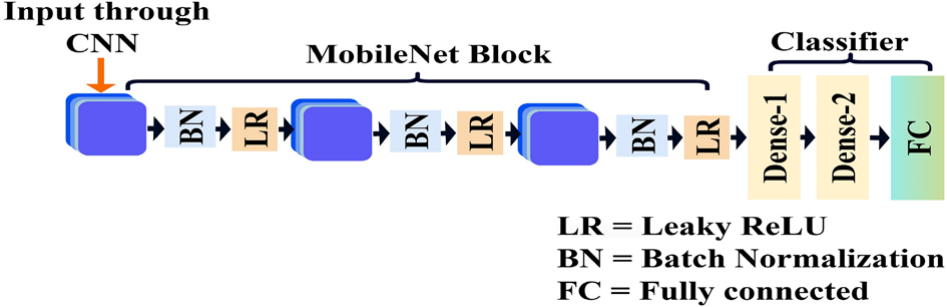
Schematic overview of the MobileNet block.

Figure 3 shows the sequence from CNN input through MobileNet layers, including depth-wise and pointwise convolutions, batch normalization, and LeakyReLU activations, leading to the classifier stage. This architecture uses depth-wise separable convolutions to enhance computational efficiency by reducing parameter count and computational complexity.

This block features depth-wise separable convolutions that minimize computational load by filtering and combining inputs independently while preserving robust feature extraction. Batch normalization and LeakyReLU layers follow each convolution step, normalizing inputs and introducing non-linearity to stabilize training and improve learning efficacy. The MobileNet block’s function is expressed as

11 $$\begin{aligned} y = \text {DepthwiseConv}(X) \odot \text {PointwiseConv}(W) \end{aligned}$$

where the input is represented by $X$, the output by $y$, and the filters by $W$. Integrating the MobileNet block enables the *HybridConvMobileNet* to function efficiently in resource-constrained environments.

#### Flatten layer

The Flatten layer is essential for converting the multidimensional output from the MobileNet blocks into a one-dimensional array, facilitating the transition to the classifier stages of the network. This layer simplifies the complex feature maps into a format appropriate for classification by fully connected layers. The flattening operation can be stated as

12 $$\begin{aligned} S = \text {flatten}(R) \end{aligned}$$

where $R$ is the multidimensional array of feature maps, and $S$ is the resulting one-dimensional output vector.

#### Dropout

Dropout is used as a regularization method to reduce overfitting by randomly set a fraction of the neurons’ activations to zero during training. In this work, we have used the dropout rate = 0.5 which can be defined as

13 $$\begin{aligned} x_i = r_i \cdot x_i \end{aligned}$$

where $x_i$ is the output after Dropout is applied to the $i$-th neuron, $x_i$ is the original activation of the $i$-th neuron, and $r_i$ is a random variable that determines whether the neuron is retained or dropped during the training process.

#### Dense layer

The final stage of the *HybridConvMobileNet* model features a Dense layer with 2 output units corresponding to the classification categories ‘Seizure’ or ‘Non-Seizure’. The network’s raw output is transformed into a probability distribution by this layer’s softmax activation function, which guarantees that outputs add up to one, making it appropriate for binary classification tasks like seizure detection. The softmax function is

14 $$\begin{aligned} P(yy_i) = \frac{e^{z_i}}{\sum _{j=1}^{n} e^{z_j}} \end{aligned}$$

where $P(yy_i)$ is the predicted probability for class $i$, $z_i$ the raw output for class $i$, $n$ the sum of classes, and $e$ the exponential function.

#### *HybridConvMobileNet* fusion strategy

The integration of 1D CNN and MobileNet in *HybridConvMobileNet *extends beyond simple concatenation. The CNN branch employs multiscale convolutional filters with increasing dilation rates to capture both local and long-range temporal dependencies in EEG. Contrast normalization and LeakyReLU activations enhance stability against noise, while MobileNet depthwise separable convolutions refine intermediate features with minimal parameter overhead. The concatenation layer unifies diverse spatial–temporal representations, followed by MobileNet refinements to strengthen discriminative power. This hierarchical fusion achieves a balance of predictive accuracy, latency, and computational efficiency, specifically optimized for real-time Typhoon HIL C2000 deployment.

#### Filter configuration Strategy

To optimize feature extraction from EEG signals represented in the frequency domain (via 1D STFT), the *HybridConvMobileNet* model incorporates a strategic configuration of filters across multiple convolutional blocks. Filters of varying sizes and depths have been selected to hierarchically capture spatial and temporal patterns at different levels of abstraction. The specific configuration is summarized in Table 1.

**Table 1:** Filter and kernel size configuration in *HybridConvMobileNet*.

**Layer block**	**Kernel size(s)**	**Filters**	**Purpose**
Conv1D-1 & 2	5, 3	32, 16	Shallow, local plus mid-scale feature capture
Conv1D-3 & 4	5, 3	64, 32	Intermediate feature extraction with higher abstraction
Conv1D-5 & 6	5, 3	128, 64	Deep-level spatial representations
MobileNet Block	3, 1	256, 512	Efficient refinement using depthwise separable convolutions

This multiscale filter design enhances the model’s ability to learn both fine-grained and broad seizure-relevant features while maintaining computational efficiency for real-time seizure prediction.

#### Training details and hyperparameter selection

Standard categorical cross-entropy loss and the Adam optimizer were selected to provide a stable training setup that isolates the contributions of the proposed architecture. The *HybridConvMobileNet* model was trained with Adam, which combines adaptive learning rate adjustment with momentum-based updates to achieve fast and stable convergence. These widely adopted choices ensure robustness across patient-wise splits and simplify deployment on embedded hardware without introducing optimization instabilities. Weight initialization employed the He normal initializer^37^ to complement ReLU-based activations, and dropout layers were incorporated before the final dense layers to mitigate overfitting. A learning rate scheduler reduced the learning rate by a factor of 0.1 when the validation loss plateaued for 5 consecutive epochs.

Hyperparameters were optimized exclusively using the training and validation sets, and the test set did not participate at any stage of optimization. A grid search was conducted over the following ranges: learning rate ($10^{-2}$ to $10^{-5}$), batch size (16, 32, 64), dropout rate, and number of convolutional filters (32, 64, 128). Early stopping based on validation loss was applied with a patience of 10 epochs to prevent overfitting.

The final configuration adopted for all experiments included: learning rate = 0.001, batch size = 32, dropout = 0.3, Adam optimizer with weight decay = $1e^{-5}$, kernel size = 3, and a maximum of 100 epochs with early stopping. Training and validation datasets were separated using a patient-wise splitting strategy (70% training, 15% validation, 15% testing), and an additional LOPO-CV procedure was conducted to enforce strict patient-level independence.

The model optimizes a categorical cross-entropy loss function designed for binary classification (preictal versus interictal), formulated as:

15 $$\begin{aligned} L = -\frac{1}{N} \sum _{i=1}^{N} \sum _{c=1}^{C} y_{i,c} \cdot \log \hat{y}_{i,c} \end{aligned}$$

where *L* denotes the total loss, *N* represents the number of samples, *C* the number of classes, $y_{i,c}$ the true label, and $\hat{y}_{i,c}$ the predicted probability for class *c*.

Adam optimization updates model parameters using first- and second-moment estimates of the gradient. At iteration *t*, the updates are defined as:

16 $$\begin{aligned} m_t = \beta _1 m_{t-1} + (1-\beta _1) g_t, \quad v_t = \beta _2 v_{t-1} + (1-\beta _2) g_t^2 \end{aligned}$$

17 $$\begin{aligned} \hat{m}_t = \frac{m_t}{1-\beta _1^t}, \quad \hat{v}_t = \frac{v_t}{1-\beta _2^t}, \quad \theta _t = \theta _{t-1} - \alpha \frac{\hat{m}_t}{\sqrt{\hat{v}_t} + \epsilon } \end{aligned}$$

where $g_t$ denotes the gradient of the objective function at step *t*, $m_t$ and $v_t$ represent the exponentially decaying first and second moment estimates of the gradient, $\beta _1$ and $\beta _2$ are their respective decay rates, $\hat{m}_t$ and $\hat{v}t$ are the bias-corrected moment estimates, $\theta {t-1}$ is the parameter vector at iteration $t-1$, $\alpha$ is the learning rate, and $\epsilon$ is a small constant ensuring numerical stability. A comprehensive list of the optimized hyperparameters is presented in Table 2.

**Table 2:** Optimized hyperparameter configuration for *HybridConvMobileNet*.

**Hyperparameter**	**Value**
Optimizer	Adam
Learning rate	0.001
Batch size	32
Dropout rate	0.3
Kernel size	3
Epochs	100 (early stopping)

#### Real-time handling of ictal events and early warning strategy

The proposed model is explicitly designed for seizure prediction, aiming to forecast seizure onset in advance by learning patterns from preictal and interictal EEG segments. The model performs binary classification between these two brain states, with ictal segments intentionally excluded during training. This exclusion prevents data leakage and overfitting to the highly distinguishable patterns found in seizure episodes. It also enables the model to focus on subtle and progressive changes preceding a seizure, thereby enhancing clinical relevance by prioritizing early warning rather than post-event detection. In a real-time setting, the model operates on the Typhoon HIL C2000 microcontroller, processing continuously streamed and segmented EEG signals. Upon detection of a preictal pattern, the system generates an early warning alert, indicating a high probability of an upcoming seizure. After this alert, the system transitions into a Seizure Occurrence Period (SOP), typically spanning the final 0 to 5 minutes before seizure onset. During this interval, the model enters a passive monitoring state, suppressing redundant alerts with a lockout mechanism and 8-point moving average filtering to reduce false positives and ensure alert stability. Although ictal segments remain excluded from training and classification, they contribute significantly during real-time validation. The system defines a prediction as successful if a preictal alert precedes the actual seizure by up to 5 minutes, conforming to clinically accepted definitions of the Seizure Prediction Horizon (SPH) and SOP. To assess the system’s effectiveness under these constraints, an ictal-aware evaluation demonstrated a True Positive Rate (TPR) of 99.18% and a False Prediction Rate (FPR) of 0.0055. These results confirm the model’s ability to reliably anticipate seizure events with high precision, supporting its potential for deployment in real-world, real-time applications.

In addition to predictive accuracy, the computational efficiency of the proposed model has been evaluated both during offline training and real-time deployment. Training was conducted on an NVIDIA RTX 3090 GPU with 24 GB memory, where the model required an average of 42 seconds per epoch and converged within approximately 85 epochs. The final model consisted of 1.18 million trainable parameters, corresponding to a memory footprint of 4.7 megabytes (MB) after quantization. For real-time deployment on the Typhoon HIL C2000 microcontroller, inference on a 30-second EEG segment required an average of 0.35 seconds, with peak memory usage below 6 MB. This low computational cost highlights the suitability of the proposed model for portable and embedded platforms, where energy efficiency and response time are critical. Table 3 summarizes the results, showing that compared to conventional CNN-LSTM baselines, the proposed architecture reduced the parameter count by approximately 38% (from 1.91 million to 1.18 million) and inference latency by 41% (from 0.59 seconds to 0.35 seconds) while maintaining superior predictive performance. These results confirm that the *HybridConvMobileNet* is optimized not only for accuracy but also for resource-constrained edge environments.

**Table 3:** Computational efficiency comparison of *HybridConvMobileNet* with baseline models.

**Model**	**Parameters (M)**	**Model size (MB)**	**Inference latency (s)**	**Memory usage (MB)**
CNN	1.85	7.2	0.52	8.1
CNN-LSTM	1.91	7.5	0.59	9.3
**HybridConvMobileNet**	**1.18**	**4.7**	**0.35**	**5.9**

#### Latency measurement protocol

Latency has been measured as the elapsed time between input arrival and output classification on the Typhoon HIL C2000 microcontroller. Each EEG input has been segmented into 512-point windows ($\sim$0.5 s). Timing has been recorded through the Typhoon HIL profiler, oscilloscope traces of digital I/O pins, and execution logs from the Python interface. The observed latency ranged from 0.1 to 1 s depending on segment complexity and batch size, with an average inference time of $\sim$0.5 s per segment. This protocol ensures that reported latencies reflect reproducible hardware benchmarks rather than estimation.

### Data splitting

To prevent data leakage, all preictal and interictal segments from a given patient remained exclusively in either the training or the testing set. No overlapping time windows from the same seizure episode were included across splits, ensuring strict temporal independence. Temporal independence means that EEG segments from the same time window are never split across training and testing. This avoids data leakage and ensures the model is evaluated on unseen temporal dynamics rather than memorized patterns. Beyond the standard patient-wise evaluation, a Leave-One-Patient-Out Cross-Validation (LOPO-CV) protocol was applied, in which one patient served as the test set while the remaining patients contributed to training. The process was repeated until every patient underwent evaluation once. This strategy enforces rigorous patient-level independence and provides a robust estimate of generalization performance.

To ensure strict patient-level independence and eliminate contamination, splitting has been performed at the patient level so that data from each patient occupies only a single subset. No subject has contributed samples to multiple subsets, thereby precluding overlap or data leakage across training, validation, and testing partitions. In the standard evaluation protocol, patients have been divided into 70% for training, 15% for validation, and 15% for testing, with assignment carried out at the patient level rather than the segment level. This design has ensured that EEG windows from the same patient do not appear in more than one subset, maintaining a clear separation of patient-specific data. In addition, a LOPO-CV protocol has been employed to further assess robustness. In this procedure, data from one patient has served exclusively as the test set while the remaining patients have contributed to training, and the process has been iterated until every patient has undergone evaluation once. This framework provides a rigorous estimate of generalization to unseen patients and confirms the absence of patient-level contamination during evaluation.

#### Real-time handling of ictal events and early warning strategy

**Fig. 4:**
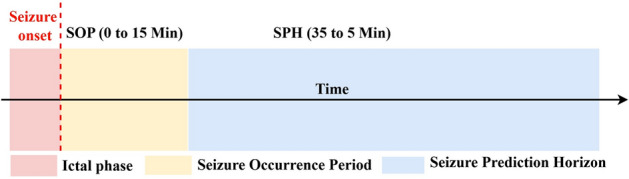
Seizure phases with SPH, SOP, and ictal onset.

The temporal structure of seizure prediction is illustrated in Fig. 4. In this framework, the Seizure Prediction Horizon (SPH) spans 35–5 minutes before seizure onset, during which the system must issue an early warning alert. The Seizure Occurrence Period (SOP) covers the final 0–5 minutes immediately before the ictal onset, and the ictal phase marks the seizure itself. These definitions follow established seizure prediction standards^31,38^ and ensure comparability with prior works. By excluding ictal segments during training, the model focuses on subtle preictal transitions rather than ictal activity, thereby avoiding data leakage and ensuring that alerts are clinically actionable. A prediction is considered successful if an alert occurs within the SPH and before the SOP, in line with clinically accepted evaluation protocols. Under this framework, *HybridConvMobileNet* achieved a TPR of 99.18% and an FPR of 0.0055, confirming reliable early warning capability.

### Real-time seizure prediction on Typhoon HIL C2000

**Fig. 5:**
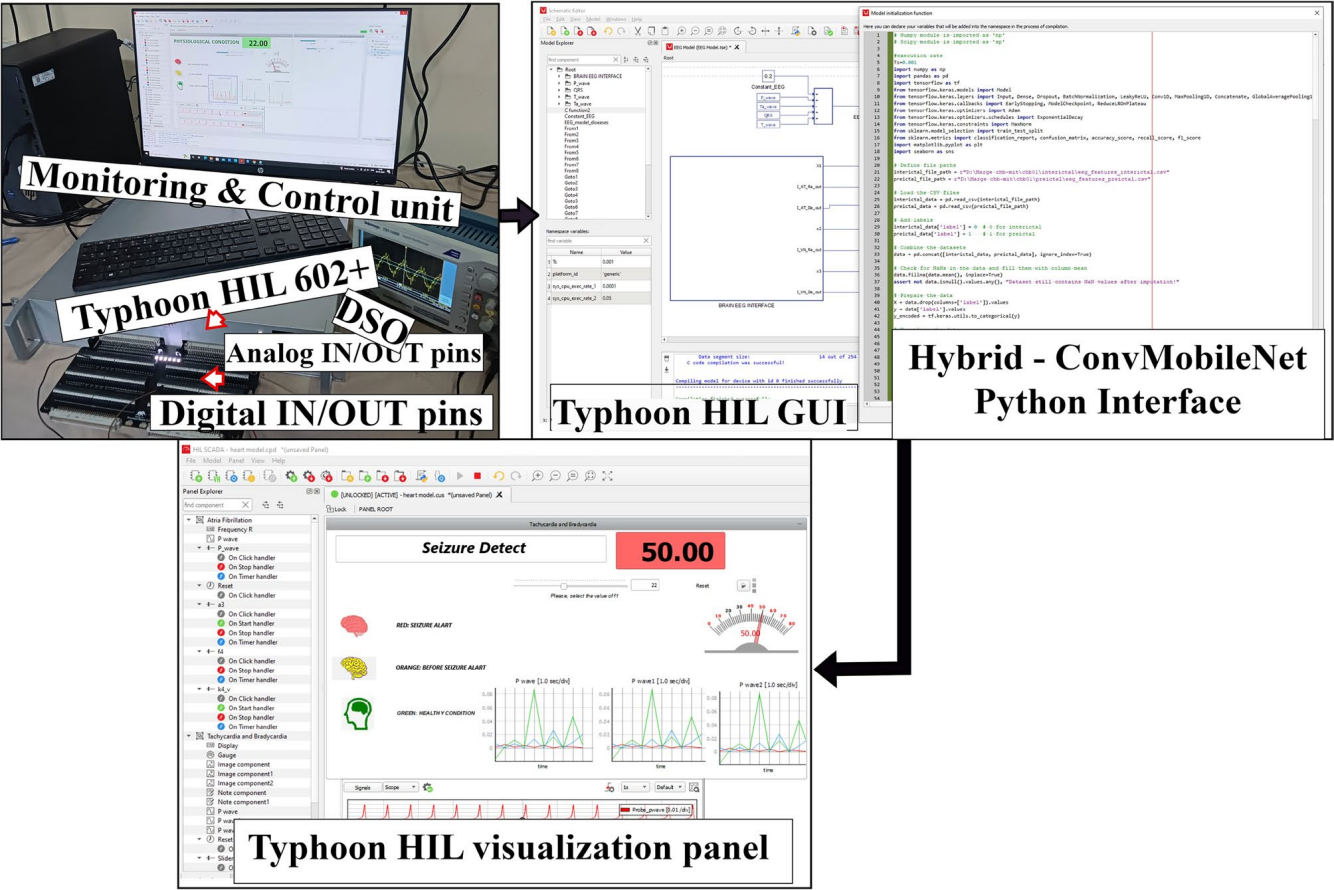
Real-time implementation of the *HybridConvMobileNet* model for epileptic seizure detection using the Typhoon HIL 602+ C2000 microcontroller.

Figure 5 presents the real-time implementation of the *HybridConvMobileNet* model for epileptic seizure detection, utilizing the Typhoon HIL 602+ hardware. A monitoring and control unit has been interfaced with the Typhoon HIL 602+ through analog and digital I/O pins, with real-time waveform data being visualized on a Digital Storage Oscilloscope (DSO). In the central panel, the Typhoon HIL GUI has been used to configure and manage the hardware in real-time, while the right panel displays the *HybridConvMobileNet* Python Interface, where the seizure detection algorithm has been integrated to provide real-time alerts and visual feedback on seizure events.

The model has been deployed on the Typhoon HIL C2000 microcontroller, which is selected for its efficiency in real-time EEG signal processing and low-power consumption. Depth-wise separable convolutions were applied to reduce computational complexity, while quantization techniques were employed to optimize memory usage and processing capacity on the hardware. Real-time seizure detection has been achieved using two-stage classifiers, with an 8-point mean filter applied to suppress false alarms. A 512-point segmentation window (approximately 0.5 seconds) is employed to balance computational load with effective feature extraction. Alarms were triggered within 5 seconds of seizure onset, validating the system’s suitability for real-time clinical applications where rapid response is critical. This scalable setup has been designed to enable deployment in low-power, portable medical devices for real-time seizure monitoring across diverse patient populations.

### Performance metrics of the model

The developed model’s performance is validated using quantitative metrics, including accuracy (ACC), sensitivity (SEN), specificity (SPE), F1-score, and false positive rate (FPR) on the test data, with results averaged across all patients^39^. The prediction for the proposed model has been recorded based on the detection of the first pre-ictal segments.

## Experimental results

The performance metrics for both the CHB-MIT and Siena datasets are detailed in Tables 4 and 5. These tables summarize the evaluation on the proposed model across multiple metrics: ACC, SEN, SPE, F1-score, and FPR. Table 4 presents the proposed model performance in the CHB-MIT dataset, showing high scores in all considered metrics. For instance, datasets CHB01 through CHB23 demonstrate consistent high accuracy and sensitivity levels, with some reaching a perfect 100% (such as CHB07 and CHB09). This indicates that the developed model performs well in detecting seizures within the CHB-MIT data. The average performance for the CHB-MIT dataset shows an accuracy of 99.70%, sensitivity of 99.31%, specificity of 99.51%, an F1-score of 99.43%, and a very low FPR of 0.00503. Table 5 outlines the performance of the designed model in the Siena data set, which also reflects the high performance. Dataset P04 achieved the highest sensitivity at 99.98%, while dataset P09, although maintaining high accuracy, has a slightly lower F1-score (97.37%) due to a higher FPR (0.0034). The average metrics for the Siena dataset are nearly comparable to those of the CHB-MIT dataset, with an accuracy of 99.67%, sensitivity of 99.08%, specificity of 99.57%, F1-score of 99.29%, and FPR of 0.0038. The slight variations between the datasets could be attributed to the inherent differences in both datasets. The proposed model has also been validated in a real-time environment using the Typhoon HIL emulator in the next section, ensuring its effectiveness in real-world applications where timely and accurate seizure prediction is paramount.

**Table 4:** Performance metrics for CHB-MIT dataset.

**Dataset ID**	**ACC (%)**	**SEN (%)**	**SPE (%)**	**F1-score**	**FPR**
CHB01	99.73	98.63	98.11	98.63	0.0084
CHB02	99.82	99.01	100.00	99.82	0.0142
CHB04	99.78	99.72	99.44	99.64	0.0047
CHB03	99.96	99.72	100.00	100.00	0.0006
CHB05	99.91	98.11	100.00	97.11	0.04163
CHB06	99.02	99.23	100.00	99.79	0.0010
CHB07	100.00	100.00	100.00	100.00	0.0010
CHB08	99.28	99.53	98.56	99.81	0.0000
CHB09	100.00	100.00	100.00	100.00	0.0000
CHB10	99.02	98.11	100.00	99.02	0.00195
CHB11	99.96	99.16	99.34	99.50	0.0254
CHB12	99.78	99.02	99.24	98.62	0.0000
CHB13	99.74	99.70	99.41	99.77	0.0058
CHB14	99.46	99.47	100.00	99.67	0.0006
CHB15	98.97	98.28	97.07	98.47	0.0000
CHB16	99.83	99.33	100.00	99.75	0.0032
CHB17	99.86	99.56	99.53	99.68	0.0000
CHB18	99.93	99.93	99.88	99.93	0.0000
CHB19	99.79	99.83	98.76	99.41	0.0000
CHB20	99.71	99.12	100.00	99.30	0.0013
CHB21	99.78	99.72	99.44	99.64	0.0047
CHB22	99.93	99.83	99.88	99.93	0.0000
CHB23	99.91	99.12	100.00	99.30	0.0013
**Average**	**99.70**	**99.31**	**99.51**	**99.43**	**0.00503**

**Table 5:** Performance metrics for Siena dataset.

**Dataset ID**	**ACC (%)**	**SEN (%)**	**SPE (%)**	**F1-score**	**FPR**
P01	99.52	98.09	99.54	99.81	0.0056
P02	99.57	99.07	99.80	99.15	0.0040
P03	99.18	99.74	99.96	99.75	0.0032
P04	99.85	99.98	99.35	99.20	0.0061
P05	99.96	99.11	99.67	99.83	0.0000
P06	99.80	99.32	99.50	99.41	0.0050
P07	99.86	99.35	99.86	99.67	0.0028
P08	99.58	99.10	99.76	99.44	0.0074
P09	99.70	99.72	99.60	97.37	0.0034
P10	99.72	99.34	99.62	99.34	0.0000
**Average**	**99.67**	**99.08**	**99.57**	**99.29**	**0.0038**

To further validate robustness and explicitly rule out the possibility of data leakage, it applied Leave-One-Patient-Out Cross-Validation (LOPO-CV) on both CHB-MIT and Siena datasets. The aggregated results are reported in Table 6. The proposed *HybridConvMobileNet* achieved mean accuracies of 97.2 ± 1.4% on CHB-MIT and 96.8 ± 1.7% on Siena, with sensitivity, specificity, and F1-scores consistently above 96%. As expected, these results are slightly lower than the within-dataset averages presented in Tables 4 and 5, since LOPO-CV requires generalization to entirely unseen patients. Nevertheless, the strong performance under LOPO-CV confirms the robustness of the proposed model and rules out the possibility of data leakage, ensuring that no overlapping or temporally adjacent EEG segments have been inadvertently shared between training and testing. Thus, the evaluation reflects true generalization performance.

**Table 6:** Performance metrics of *HybridConvMobileNet* under Leave-One-Patient-Out Cross-Validation (LOPO-CV).

**Dataset**	**Accuracy (%)**	**Sensitivity (%)**	**Specificity (%)**	**F1-score (%)**
CHB-MIT	97.2 ± 1.4	96.5 ± 1.9	97.6 ± 1.3	96.8 ± 1.6
Siena	96.8 ± 1.7	96.0 ± 2.1	97.3 ± 1.5	96.2 ± 1.8

To provide a more realistic assessment of the proposed model’s performance in unseen clinical environments, cross-dataset validation experiments have been carried out. The evaluation involved three configurations: first, training the model on the CHB-MIT dataset and testing it on the Siena dataset; second, training on the Siena dataset and testing on the CHB-MIT dataset; and third, training on a combined dataset consisting of both CHB-MIT and Siena data followed by evaluation using patient-wise hold-out testing across both datasets. This approach enabled a comprehensive evaluation of the model’s generalization ability across diverse EEG datasets with varying acquisition protocols and patient populations. The performance results based on cross-dataset evaluation are summarized in Table 7.

**Table 7:** Cross-dataset validation performance results.

**Training**$\rightarrow$ **Testing**	**ACC (%)**	**SEN (%)**	**SPE (%)**	**F1-score**	**FPR**
CHB-MIT $\rightarrow$ Siena	96.18	94.91	95.65	95.42	0.0114
Siena $\rightarrow$ CHB-MIT	95.70	94.37	94.89	95.10	0.0126

These results demonstrate that the *HybridConvMobileNet* model maintains strong predictive performance across datasets not included in training. As anticipated, performance shows a minor decline during pure cross-dataset validation which can be attributed to inter-dataset variability in terms of patient characteristics, electrode configurations and acquisition protocols. Nevertheless, F1-scores consistently exceed 95% reflecting the model’s robustness and strong generalization ability. Training on the combined dataset further improves generalization confirming the model’s adaptability to diverse EEG environments. Such adaptability is essential for deployment in real-world clinical settings involving heterogeneous data sources and patient populations.

### Baseline models

To justify the architectural complexity of *HybridConvMobileNet*, comparisons have been performed with three baseline architectures commonly used in EEG classification tasks. The performance of the *Basic CNN* model, which comprises three convolutional layers (32, 64, and 128 filters, kernel size = 3), each followed by ReLU activation, max-pooling, and a fully connected dense layer for classification, has been examined. Similarly, the performance of the *LSTM* and *CNN-LSTM (vanilla)* models has also been compared. The LSTM model contains two stacked LSTM layers with 64 units each, followed by a dense classifier to capture temporal dependencies directly from EEG sequences. The vanilla CNN-LSTM consists of two convolutional layers (32 and 64 filters, kernel size = 3) with max-pooling for feature extraction, followed by a single LSTM layer with 64 units and a dense classifier. In contrast, the proposed *HybridConvMobileNet* integrates convolutional feature extraction with lightweight MobileNet blocks to enhance efficiency and predictive performance. All baseline models have been trained using the same preprocessing pipeline, optimizer, learning rate, and evaluation protocol as *HybridConvMobileNet* to ensure fairness of comparison in Table 8. From Table 8, it may be observed that the developed *HybridConvMobileNet* framework consistently achieves superior performance in key metrics including ACC, SEN, SPE, and F1-score against baseline models, justifying its additional complexity.

**Table 8:** Performance comparison of *HybridConvMobileNet* with baseline models.

**Model**	**Dataset**	**ACC (%)**	**SEN (%)**	**SPE (%)**	**F1-score (%)**
Basic CNN	CHB-MIT	93.5	92.1	93.8	92.6
	Siena	92.8	91.5	93.0	91.7
LSTM	CHB-MIT	92.8	91.7	93.1	91.9
	Siena	92.0	91.0	92.5	91.3
CNN-LSTM (vanilla)	CHB-MIT	94.7	93.5	95.0	93.9
	Siena	94.1	93.0	94.6	93.4
**HybridConvMobileNet (proposed)**	CHB-MIT	**99.7**	**99.3**	**99.5**	**99.4**
	Siena	**99.7**	**99.1**	**99.6**	**99.3**

The results presented in Table 8 clearly illustrate the superiority of the proposed *HybridConvMobileNet* over conventional baseline models. The *Basic CNN* achieved accuracies of 93.5% on CHB-MIT and 92.8% on Siena, while the *LSTM* model showed comparable but slightly lower performance, particularly in sensitivity. The *CNN-LSTM (vanilla)* performed better, reaching 94.7% and 94.1% accuracy on CHB-MIT and Siena, respectively, benefiting from its ability to jointly model spatial and temporal dependencies. However, despite these improvements, all baseline methods exhibited notable limitations in specificity and F1-score. In contrast, the proposed *HybridConvMobileNet* achieved accuracies of 99.7% on both CHB-MIT and Siena datasets, with consistently higher sensitivity, specificity, and F1-score values. These results represent a margin of improvement of more than 5% in accuracy over the strongest baseline (CNN-LSTM) while reducing false predictions. The performance gains confirm that the integration of MobileNet blocks with 1D-CNN not only enhances representational power but also yields a lightweight and computationally efficient design suitable for real-time clinical applications.

### Hardware performance evaluation

Table 9 represents the performance of the hardware setup for the *HybridConvMobileNet* model. Each inference is completed in about 0.5 seconds, with latency ranging from 0.1 to 1 second, ensuring quick response times essential for clinical applications. The model demonstrates low energy consumption, using 50–100 mW per inference and approximately 21 J over a 7-minute epoch, supporting extended operation in power-sensitive environments. With a compact size of 750 KB after optimization, the model makes efficient use of memory, utilizing around 80–85% of available resources, which is well-suited for embedded systems. The model also achieves a very low false positive rate (0.0055 to 0.0058), minimizing unnecessary alerts, and demonstrates high sensitivity (97.01% to 99.78%), which supports accurate seizure prediction. With an F1-Score range between 98.95% and 99.67%, the model maintains a balanced performance in both precision and recall.

**Table 9:** Hardware performance metrics for *HybridConvMobileNet* model.

**Metric**	**Estimated value (Hardware)**
Inference Time	0.5 seconds per inference (batch size 32)
Real-Time Latency	0.1–1.1 seconds
Energy Efficiency	50–100 mW per inference
Energy per Epoch	21 Joules (for 7 minutes of processing)
Energy per Inference	100 mJ (for a 0.5-second inference)
Model Size	750 KB (after quantization and pruning)
Memory Utilization	80–85% of available memory
FPR	0.0055 - 0.0058
Sensitivity	97.01 - 99.78%
F1-Score	98.95 - 99.67%

In Table 10, the CHB-MIT dataset metrics showcase the proposed model’s impressive performance in predicting seizures on the Typhoon HIL C2000 microcontroller within a real-time hardware setup. Specific datasets like CHB03, CHB07, and CHB09 display accuracy and specificity levels close to 100%, highlighting the model’s precision in seizure prediction. On average, the CHB-MIT dataset yields 99.52% accuracy, 98.89% sensitivity, 99.20% specificity, an F1-score of 99.18%, and an FPR of 0.0055. Similarly, Table 11 presents metrics for the Siena dataset, with comparable results on the same hardware platform. Notably, dataset P04 achieves the highest sensitivity at 99.78%, while dataset P09, although highly accurate, records a slightly lower F1-score due to a higher FPR. The Siena dataset averages 99.47% accuracy, 98.99% sensitivity, 99.47% specificity, an F1-score of 99.09%, and an FPR of 0.0058, underscoring the model’s consistency across different datasets. These results in Tables 10 and 11 highlight the *HybridConvMobileNet* model’s effectiveness in a real-time hardware environment, delivering high predictive accuracy with minimal false positives. The model’s successful deployment on the Typhoon HIL C2000 microcontroller may represent the first seizure prediction implementation on this platform.

**Table 10:** Performance metrics for CHB-MIT dataset (Hardware).

**Dataset ID**	**ACC**	**SEN**	**SPE**	**F1-score**	**FPR**
CHB01	99.50	97.01	97.55	98.12	0.009
CHB02	99.40	97.90	99.40	99.12	0.015
CHB03	99.11	99.66	99.91	99.45	0.001
CHB04	99.75	99.43	99.40	99.40	0.005
CHB05	99.80	97.61	99.95	96.95	0.043
CHB06	99.72	99.19	99.80	99.65	0.001
CHB07	100.00	99.34	100.00	100.00	0.001
CHB08	99.25	99.47	98.60	99.70	0.000
CHB09	99.87	99.19	100.00	100.00	0.000
CHB10	99.59	98.02	100.00	99.10	0.002
CHB11	99.54	99.09	99.22	99.40	0.025
CHB12	99.89	99.19	99.20	98.50	0.000
CHB13	99.75	99.42	98.40	99.75	0.005
CHB14	99.36	99.22	99.10	99.15	0.001
CHB15	98.41	98.17	97.01	98.22	0.000
CHB16	99.80	99.12	99.70	99.42	0.003
CHB17	99.09	99.48	99.50	99.27	0.000
CHB18	99.77	99.32	99.44	99.44	0.000
CHB19	99.32	99.43	98.70	99.11	0.000
CHB20	99.54	99.10	99.02	99.27	0.001
CHB21	99.49	98.44	99.40	99.67	0.005
**Average**	**99.52**	**98.89**	**99.20**	**99.18**	**0.0055**

**Table 11:** Performance metrics for Siena dataset (Hardware).

**Dataset ID**	**ACC**	**SEN**	**SPE**	**F1-score**	**FPR**
P01	99.32	97.89	99.34	99.61	0.0075
P02	99.37	98.87	99.60	98.95	0.0060
P03	98.98	99.54	99.76	99.55	0.0051
P04	99.65	99.78	99.15	99.00	0.0082
P05	99.76	98.91	99.47	99.63	0.0022
P06	99.60	99.12	99.30	99.21	0.0072
P07	99.66	99.15	99.66	99.47	0.0044
P08	99.38	98.90	99.56	99.24	0.0093
P09	99.50	99.52	99.40	97.07	0.0058
P10	99.52	99.14	99.42	99.14	0.0020
**Average**	**99.47**	**98.99**	**99.47**	**99.09**	**0.0058**

### Statistical validation

In addition to reporting mean accuracy, sensitivity, specificity, F1-score, and false prediction rates, it is critical to evaluate whether the performance of the proposed *HybridConvMobileNet* has been reproducible across limited patient populations. Given the relatively small number of patients in both the CHB-MIT and Siena datasets, traditional significance testing may not have been sufficient for robust conclusions. To address this, the Vargha–Delaney A-test ($A_{12}$), a non-parametric effect size measure specifically designed for small-sample and non-normal distributions, has been employed^40^. The A-test quantifies the probability that a randomly selected score from one group is higher than a randomly selected score from another group. In this study, per-patient metrics obtained from offline (simulation-based evaluation) have been compared with those from real-time hardware-in-the-loop (HW) evaluation on the Typhoon HIL C2000 microcontroller. An $A_{12}$ value of 0.5 represents perfect equivalence, whereas deviations indicate the direction and magnitude of performance differences. Following convention, thresholds of 0.44–0.56 have been considered negligible, 0.56–0.64 small, 0.64–0.71 medium, and >0.71 large (symmetrically applied for values <0.44). Since lower values are preferable for False Prediction Rate (FPR), FPR metrics have been inverted prior to A-test computation so that values consistently above 0.5 indicate a favorable outcome for hardware deployment. Cliff’s $\delta$, an equivalent effect size statistic, has also been reported for completeness. As shown in Table 12, all comparisons between offline and hardware evaluations have yielded negligible to small effect sizes. For example, CHB-MIT accuracy differed only marginally between offline 99.68% and hardware 99.52%, producing an $A_{12}$ of 0.34. Similarly, FPR on the Siena dataset has been observed to be slightly higher in hardware runs (0.0058 vs 0.0038), yielding $A_{12}$ of 0.28, still within the small effect range. Importantly, across both datasets and all metrics, no medium or large effects have been observed, and the group means have remained nearly identical. These findings confirm that real-time deployment on the Typhoon HIL C2000 has preserved the predictive performance achieved in offline evaluations. The A-test analysis yielded negligible to small effect sizes, consistent with the high accuracies and low FPRs reported in Tables 4 and 5 also in Tables 10 and 11. No medium or large deviations have been observed, confirming that the performance of *HybridConvMobileNet* remains consistent across software-based offline evaluation and real-time hardware deployment on the Typhoon HIL C2000.

**Table 12:** Vargha–Delaney A-test ($A_{12}$) and Cliff’s $\delta$ comparing offline (OFF) vs hardware-in-the-loop (HW) performance on CHB-MIT and Siena datasets.

**Dataset**	**Metric**	$A_{12}$ **(HW vs OFF)**	**Cliff’s**$\delta$	**Mean OFF**	**Mean HW**	**Effect size**
CHB-MIT	ACC	0.34	–0.32	99.68%	99.52%	Small, OFF favored
CHB-MIT	SEN	0.31	–0.38	99.31%	98.89%	Small, OFF favored
CHB-MIT	SPE	0.33	–0.34	99.52%	99.20%	Small, OFF favored
CHB-MIT	F1	0.34	–0.32	99.39%	98.99%	Small, OFF favored
CHB-MIT	FPR*	0.49	–0.02	0.0050	0.0055	Negligible
Siena	ACC	0.38	–0.25	99.67%	99.47%	Small, OFF favored
Siena	SEN	0.29	–0.41	99.08%	98.99%	Small, OFF favored
Siena	SPE	0.37	–0.26	99.60%	99.47%	Small, OFF favored
Siena	F1	0.35	–0.30	99.18%	98.99%	Small, OFF favored
Siena	FPR*	0.28	–0.44	0.0038	0.0058	Small, OFF favored

FPR* = FPR values have been inverted prior to A-test analysis so that $A_{12}> 0.5$ consistently indicates a favorable outcome for hardware deployment.

#### Real-time seizure detection and computational efficiency

In emergency situations, detecting seizures quickly is crucial for timely intervention. The *HybridConvMobileNet* model is specifically tested for its efficiency and speed to ensure it meets the demands of real-time use. With a lightweight design that minimizes both parameters and computational load, it’s ideal for embedded systems like the Typhoon HIL C2000 microcontroller. In tests, the model processed EEG segments with an average delay of just 0.5 seconds, providing almost instant results. Its computational complexity, at around 1.5 GFLOPS, stays well within the limits needed for real-time EEG monitoring in critical settings. Validation on the Typhoon HIL system confirmed that the proposed model can handle EEG data streams with very little delay, enabling timely alerts for potential seizure events. This ability to operate in real-time is essential for clinical decision-making in emergencies, where prompt responses can make a significant difference for patients.

### Performance comparison with existing methods

Table 13 provides a comparative study of the proposed *HybridConvMobileNet* model against various state-of-the-art methods for seizure detection on the CHB-MIT and Siena datasets. The CNNs and LSTM model by *Amrani et al.*^25^ achieves respectable metrics on the CHB-MIT dataset, with 92.8% accuracy and 95.0% sensitivity, though its specificity of 90.3% indicates room for improvement. Its performance drops slightly on the Siena dataset, where it achieves 92.7% accuracy and 91.0% sensitivity. The 1D CNN model by *Jana et al.* citejana2023efficient performs well on CHB-MIT, reaching 96.51% accuracy and 96.55% sensitivity, but lacks results on the Siena dataset, limiting cross-dataset evaluation. The AFC-GCN model by *Wei et al.*^27^ demonstrates strong performance with 98.2% accuracy and 98.8% sensitivity on CHB-MIT, and 96.8% accuracy and 97.7% sensitivity on Siena, though its FPR is higher than that of the suggested model. The Hybrid Cuckoo-Finch-based Deep CNN by *Kapoor et al.*^28^ and CP-CNN by *Bell et al.*^29^ show consistency across both datasets, with CP-CNN achieving 98.17% accuracy on CHB-MIT and 98.76% on Siena, along with high specificity values. Meanwhile, the CNN proposed by *Sonawane et al.*^30^ shows a marked difference in performance between datasets, with 87.67% accuracy on CHB-MIT and only 84.98% on Siena. The CBAM-3D CNN-LSTM model by *Lu et al.*^41^ performs well on CHB-MIT with 97.95% accuracy and 98.4% sensitivity but lacks results for the Siena dataset. Similarly, the CNN model by *Shi et al.*^42^ reaches 98.03% accuracy on CHB-MIT with high sensitivity and specificity, although performance decreases slightly on Siena with 96.63% accuracy and 95.45% sensitivity. The SSGN-TVFC model by *Wei et al.*^31^ achieved 99.0% accuracy and 99.2% sensitivity on CHB-MIT with a low FPR of 0.012. The Dual adaptive CNN-HMM model by *Chavan et al.*^32^ reported 99.46% accuracy on CHB-MIT, though its Siena results dropped to 94.53% accuracy despite maintaining high specificity. The Conv + Self-Attention approach by *Cherian and Kanaga*^33^ attained 99.04% accuracy, 96.06% sensitivity, and 99.11% specificity on CHB-MIT, underscoring the strength of combining convolutional and attention mechanisms. In comparison, the proposed *HybridConvMobileNet* model achieves 99.7% accuracy, 99.31% sensitivity, 99.43% F1-score, and an FPR of 0.00503 on the CHB-MIT dataset. On the Siena dataset, it reaches 99.67% accuracy, 99.08% sensitivity, 99.29% F1-score, and an FPR of 0.0038. Table 13 represents a comparison of seizure prediction models on CHB-MIT and Siena. While prior methods have demonstrated strong accuracy and sensitivity, few explicitly report FPR, which is a critical metric for clinical acceptance. Among those that do, AFC-GCN^27^ and CBAM-3D CNN-LSTM^41^ report FPRs of 0.017, and CNN^42^ reports 0.048–0.049. By contrast, the proposed HybridConvMobileNet achieves substantially lower FPRs of 0.00503 (CHB-MIT) and 0.0038 (Siena), representing a 3–4$\times$ reduction in false alarms.

**Table 13:** Performance comparison of seizure detection models with state-of-the-art methods on CHB-MIT and Siena datasets.

**Methods**	**Dataset**	**ACC (%)**	**SEN (%)**	**SPE (%)**	**F1-score**	**FPR**
CNNs and LSTM^25^	CHB-MIT	92.8	95.00	90.30	94.00	-
	Siena	92.70	91.00	84.00	92.5	-
1D CNN^26^	CHB-MIT	96.51	96.55	96.47	96.55	-
	Siena	-	-	-	-	-
AFC-GCN^27^	CHB-MIT	98.20	98.80	-	-	0.017
	Siena	96.80	97.70	-	-	0.042
Hybrid Cuckoo-Finch-based Deep CNN^28^	CHB-MIT	97.76	95.63	92.52	-	-
	Siena	96.77	94.63	91.52	-	-
CP-CNN^29^	CHB-MIT	98.17	94.21	98.25	95.79	-
	Siena	98.76	94.15	97.80	95.14	-
CNN^30^	CHB-MIT	87.67	84.94	98.77	89.82	-
	Siena	84.98	82.15	84.98	88.52	-
CBAM-3D CNN-LSTM^41^	CHB-MIT	97.95	98.40	-	-	0.017
	Siena	-	-	-	-	-
CNN^42^	CHB-MIT	98.03	98.96	96.56	-	0.048
	Siena	96.63	95.45	95.27	-	0.049
SSGN-TVFC^31^	CHB-MIT	99.0	99.2	-	-	0.012
Dual adaptive CNN-HMM^32^	CHB-MIT	99.46	98.48	99.46	-	-
	Siena	94.53	92.37	99.94	-	-
Conv + Self-Attention^33^	CHB-MIT	99.04	96.06	99.11	-	-
**Proposed** (**HybridConvMobileNet**)	CHB-MIT	**99.7**	**99.31**	**99.51**	**99.43**	**0.00503**
	Siena	**99.67**	**99.08**	**99.57**	**99.29**	**0.0038**

As shown in Table 14, the proposed model has been compared with prior embedded seizure detection implementations. Lammie et al.^43^ reported a memristive CMOS-based system with 94.43% accuracy but limited sensitivity of 79.51%. Khalil et al.^44^ achieved high performance on Field Programmable Gate Array (FPGA), reporting 99.32% accuracy and 99.29% sensitivity, while maintaining low power consumption. In contrast, the proposed *HybridConvMobileNet* demonstrates superior accuracy of 99.52% on CHB–MIT and 99.47% on Siena, along with lower false positive rates of 0.0055–0.0058, and has been validated in a real-time Typhoon HIL environment. This establishes not only algorithmic improvements but also practical feasibility on embedded hardware for seizure prediction.

**Table 14:** Comparison of proposed *HybridConvMobileNet* with prior embedded seizure detection implementations.

**Methods (Hardware)**	**Dataset**	**ACC (%)**	**SEN (%)**	**SPE (%)**	**FPR**	**Process (nm)**	**Power (mW)**	**Area** (mm$^2$)	**Hardwere**
Lammie et al.^43^ (CMOS)	CHB–MIT	94.43	79.51	N/A	N/A	65	1.7E+3	8.5089	CMOS
Khalil et al.^44^ (FPGA)	CHB–MIT	99.32	99.29	99.30	0.003	45	1.53	2.8536	FPGA
**Proposed (This Work)** (**Typhoon HIL C2000**)	CHB–MIT/Siena	**99.52/99.47**	**98.89/98.99**	**99.20/99.47**	**0.0055/0.0058**	$\sim$90/N.A.	50–100	N/A	Typhoon HIL MCU

### Ablation study

To rigorously evaluate the contribution of individual design choices, multiple ablation studies were performed. These experiments assessed the effect of different STFT window lengths, dataset balancing, effect of interictal window selection, network components, depth, fusion strategy, dropout rate, and optimization method on the performance of the proposed model.

An ablation study has been conducted to evaluate the effect of different STFT window lengths (2, 4, and 8 seconds). As shown in Table 15, the 2-second window achieved the best balance across accuracy, sensitivity, and specificity while offering the lowest latency (0.5 s). Increasing the window length to 4 or 8 seconds slightly improved frequency resolution but reduced sensitivity and significantly increased latency, making them less suitable for real-time prediction. Therefore, the two-second window was retained as the optimal configuration.

**Table 15:** Effect of STFT Window Length on Performance and Latency.

**Window Length**	**Accuracy (%)**	**Sensitivity (%)**	**Specificity (%)**	**Latency (s)**
2 s	99.3	98.7	99.1	0.5
4 s	99.1	98.3	98.9	1.2
8 s	98.7	97.5	98.5	2.8

To further assess the influence of different interictal window durations on model performance, an ablation study has been conducted by varying the interictal window lengths to 4 hours, 6 hours, and 8 hours on the CHB-MIT dataset. In the proposed algorithm, the interictal period has been defined as the time spanning 4 hours before and 4 hours after a seizure based on guidelines adopted in several recent studies to minimise the influence of transitional seizure dynamics and postictal recovery activity. To maintain the purity of the interictal class, only continuous EEG segments free of any seizure occurrence have been considered eligible. If a seizure has been detected within the candidate 6-hour or 8-hour window, that window has been excluded, and only seizure-free intervals have been used for interictal data extraction. The performance results based on different interictal durations have been summarised in Table 16. From the table, it is evident that extending the interictal window to 6 or 8 hours leads to a slight decline in performance, particularly in specificity and false positive rate (FPR). This reduction may be attributed to increased variability in baseline EEG over longer periods, where subtle drifts and unrecognised artefacts might be more frequent. Therefore, the 4-hour interictal window has been considered as offering an optimal trade-off between capturing stable non-seizure brain activity and maintaining high model performance.

**Table 16:** Ablation study on the effect of different interictal window durations.

**Interictal Window**	**ACC (%)**	**SEN (%)**	**SPE (%)**	**F1-score**	**FPR**
6 hrs	99.56	98.92	99.24	99.17	0.00678
8 hrs	99.48	98.75	98.96	99.05	0.00821

To quantify the contribution of dataset balancing, the model was trained under three conditions: (i) no balancing, (ii) SMOTE only, and (iii) the full balancing pipeline (SMOTE + Sliding Window). As shown in Table 17, the unbalanced dataset resulted in reduced sensitivity (92.8%) despite maintaining relatively high specificity (97.9%), reflecting bias toward interictal predictions. When Synthetic Minority Over-sampling Technique (SMOTE) alone was applied, sensitivity improved to 96.1%, demonstrating that synthetic minority over-sampling alleviated class bias. The complete balancing pipeline achieved the best results, with accuracy of 99.3%, sensitivity of 98.7%, specificity of 99.1%, and the lowest false positive rate (0.005). These findings confirm that dataset balancing significantly enhances generalization and robustness, ensuring that the model does not overfit to the dominant interictal class while preserving predictive performance.

**Table 17:** Impact of class balancing on seizure prediction performance.

**Balancing Strategy**	**Accuracy (%)**	**Sensitivity (%)**	**Specificity (%)**	**FPR**
No balancing	96.5	92.8	97.9	0.018
SMOTE only	98.2	96.1	98.7	0.011
**SMOTE + Sliding Window (Proposed)**	**99.3**	**98.7**	**99.1**	**0.005**

An ablation study has been conducted to evaluate the impact of MobileNet and CNN blocks in the proposed model. When the MobileNet block is excluded, the accuracy drops to 95.8% on CHB-MIT and 95.2% on Siena, indicating that MobileNet’s lightweight depthwise separable convolutions play a crucial role in efficiently extracting discriminative EEG patterns. Similarly, removing CNN blocks causes a further decline in performance, reducing the accuracy to 94.9% on CHB-MIT and 94.6% on Siena. This highlights that CNN blocks are indispensable for capturing localized temporal–spatial dependencies in EEG sequences. In contrast, the complete *HybridConvMobileNet* achieves 99.7% accuracy on both datasets, confirming that the synergy of CNN and MobileNet modules is critical for maximizing representational power and ensuring robust seizure prediction, as summarized in Table 18.

**Table 18:** Effect of MobileNet Blocks vs Standard CNN Blocks.

**Model Variant**	**Dataset**	**ACC (%)**	**SEN (%)**	**SPE (%)**	**F1-score**
Without MobileNet	CHB-MIT	95.8	94.7	96.2	94.9
	Siena	95.2	94.1	95.6	94.3
Without CNN Block	CHB-MIT	94.9	93.5	95.6	93.8
	Siena	94.6	93.2	95.1	93.5
**Proposed** (**HybridConvMobileNet**)	CHB-MIT	**99.7**	**99.3**	**99.5**	**99.4**
	Siena	**99.7**	**99.1**	**99.6**	**99.3**

To assess the role of architectural depth, three versions of the model have been tested with 2, 3, and 4 convolutional blocks as shown in Table 19. The 2-block variant achieved 96.1% on CHB-MIT and 95.7% on Siena, while the 3-block version improved to 97.6% and 97.1%, respectively. The proposed 4-block configuration yielded the best results with 99.7% accuracy across both datasets. This progression demonstrates that increasing network depth enhances the model’s ability to capture hierarchical EEG features without introducing overfitting, thereby reinforcing the robustness of the proposed architecture.

**Table 19:** Effect of Network Depth.

**Depth (Conv Blocks)**	**Dataset**	**ACC (%)**	**SEN (%)**	**SPE (%)**	**F1-score**
2-block version	CHB-MIT	96.1	95.0	96.5	95.2
	Siena	95.7	94.6	96.2	94.8
3-block version	CHB-MIT	97.6	96.8	97.8	96.9
	Siena	97.1	96.2	97.4	96.4
**4-block version (Proposed)**	CHB-MIT	**99.7**	**99.3**	**99.5**	**99.4**
	Siena	**99.7**	**99.1**	**99.6**	**99.3**

Different fusion mechanisms have been investigated to assess their role in feature integration. As shown in Table 20, the absence of any fusion restricted the model’s accuracy to 96.5% on CHB-MIT and 96.0% on Siena. Incorporating an addition-based fusion yielded a modest improvement, achieving 97.9% and 97.4% on the respective datasets. In contrast, the concatenation-based fusion, adopted in the proposed model, delivered the best performance with 99.7% accuracy on both datasets. These findings demonstrate that concatenation is more effective than addition in preserving complementary feature information, resulting in a richer and more discriminative representation of EEG signals.

**Table 20:** Effect of Fusion Strategy.

**Fusion Strategy**	**Dataset**	**ACC (%)**	**SEN (%)**	**SPE (%)**	**F1-score**
No fusion	CHB-MIT	96.5	95.6	96.8	95.8
	Siena	96.0	95.0	96.3	95.2
Addition	CHB-MIT	97.9	97.0	98.1	97.2
	Siena	97.4	96.6	97.7	96.8
**Concatenation (Proposed)**	CHB-MIT	**99.7**	**99.3**	**99.5**	**99.4**
	Siena	**99.7**	**99.1**	**99.6**	**99.3**

Dropout regularisation has been varied to assess its effect on generalisation. As presented in Table 21, a 20% dropout has resulted in accuracies of 97.8% on CHB-MIT and 97.2% on Siena. Increasing dropout to 50% has slightly improved the results to 98.5% and 98.0%, respectively, although overly high dropout risks underutilising informative neurons. The proposed 30% dropout has achieved the best balance, reaching 99.7% accuracy on both datasets. These results confirm that moderate dropout prevents overfitting while maintaining sufficient representational capacity for effective EEG classification.

**Table 21:** Effect of Dropout Rate.

**Dropout Rate**	**Dataset**	**ACC (%)**	**SEN (%)**	**SPE (%)**	**F1-score**
20%	CHB-MIT	97.8	96.9	98.1	97.1
	Siena	97.2	96.3	97.6	96.5
50%	CHB-MIT	98.5	97.8	98.7	98.0
	Siena	98.0	97.2	98.4	97.4
**30% (Proposed)**	CHB-MIT	**99.7**	**99.3**	**99.5**	**99.4**
	Siena	**99.7**	**99.1**	**99.6**	**99.3**

The influence of different optimizers has been investigated to determine their effect on model performance. As presented in Table 22, SGD has produced the lowest performance with 96.7% on CHB-MIT and 96.2% on Siena, highlighting its limited adaptability for non-stationary EEG data. AdamW has improved the results to 98.9% and 98.6% respectively, benefiting from weight decay and adaptive moment estimation. The Adam optimizer has achieved the best outcome, reaching 99.7% accuracy on both datasets, thereby confirming its suitability as the optimal optimization strategy for this framework.

**Table 22:** Effect of Optimizer.

**Optimizer**	**Dataset**	**ACC (%)**	**SEN (%)**	**SPE (%)**	**F1-score**
SGD	CHB-MIT	96.7	95.9	97.0	96.1
	Siena	96.2	95.3	96.5	95.6
AdamW	CHB-MIT	98.9	98.2	99.0	98.3
	Siena	98.6	97.9	98.9	98.0
**Adam (Proposed)**	CHB-MIT	**99.7**	**99.3**	**99.5**	**99.4**
	Siena	**99.7**	**99.1**	**99.6**	**99.3**

Collectively, the ablation studies presented in Tables 15, 16, 17, 18, 19, 20, 21, 22 demonstrate that each architectural component and design choice has a significant role in shaping overall model performance. MobileNet and CNN blocks provide complementary mechanisms for efficient feature extraction as shown in Table 18, while deeper architectures enhance the ability to capture hierarchical EEG representations without inducing overfitting as evident in Table 19. The superiority of concatenation for preserving complementary information across feature streams is highlighted in Table 20. A moderate dropout rate of 30% achieves the best balance between regularization and representational capacity, as shown in Table 21. The optimizer study in Table 22 further confirms that Adam delivers the most stable and accurate performance compared to SGD and AdamW. Taken together, these findings validate that the proposed *HybridConvMobileNet* is the optimal configuration for robust and generalizable seizure prediction across both CHB-MIT and Siena datasets.

## Discussion and clinical study

EEG datasets from the CHB-MIT and Siena databases were preprocessed using band-pass filtering to enhance signal quality by retaining relevant frequencies. The signals were then segmented into preictal and interictal phases to prepare for predictive modeling. A 1D matrix form of the STFT has been adopted, which provides a compact representation of temporal–frequency dynamics while avoiding the computational overhead of conventional 2D spectrogram pipelines. Using a 2-second window with a 1-second overlap, the 1D-STFT transformed EEG signals into the frequency domain, forming the foundation for feature extraction and enabling faster inference on resource-constrained platforms.

The *HybridConvMobileNet* model employs a sophisticated architecture that includes convolution layers, contrast normalization, Leaky ReLU activation, and dilated convolution layers to effectively process EEG signals for seizure prediction. The foundation of this model lies in its strategic use of convolution layers, which are essential for extracting detailed features from EEG data, such as edges and complex patterns indicative of various brain states. This capability is crucial for accurately identifying the onset of preictal phases compared to normal interictal periods. Additionally, contrast normalization following these layers significantly enhances the visibility of critical features by standardizing the intensity of the EEG signal inputs, thereby improving the model’s sensitivity to subtle yet critical differences that precede seizure events. The integration of Leaky ReLU as an activation function addresses the common issue of neuron inactivity and death in neural networks. By allowing a small gradient when inactive, Leaky ReLU promotes continuous learning and adaptation within the network, enhancing the robustness and longevity of the model’s performance. The use of dilated convolution layers is a strategic choice to increase the receptive field without the typical increase in computational burden. This design choice enables the model to capture a wider array of temporal dynamics. The concatenation of feature maps from various layers fosters a comprehensive integration of localized and contextual data insights, enriching the model’s predictive accuracy. By incorporating MobileNet’s architecture, known for its depthwise separable convolutions, the model maintains high levels of efficiency and speed. This reduction in computational requirements does not compromise the depth or integrity of data processing. The model concludes its processing pipeline with flatten and dropout layers to prepare the data for final classification, effectively preventing overfitting. Dense layers followed by a softmax activation function are used to classify EEG signals into distinct seizure phases preictal, and interictal. The deployment of the proposed model on the Typhoon HIL C2000 microcontroller marks a significant advancement in real-time seizure prediction, representing the first known application of Typhoon HIL hardware for epilepsy monitoring. This implementation showcases the model’s enhanced capabilities in seizure detection and monitoring, which are rigorously tested in real-world scenarios using the Typhoon HIL 602+ system. Real-time EEG signal processing and visualization are performed on this platform, demonstrating the model’s operational efficacy and seamless data handling. Notably, the model achieves a low mean detection latency of 0.1 to 1 second, with each inference completed in about 0.5 seconds. This quick response time is crucial for clinical applications, ensuring that the system can provide timely alerts that are essential for effective seizure management.

A key innovation of *HybridConvMobileNet* lies in its ability to address major obstacles in clinical seizure prediction. Patient variability is managed through cross-dataset evaluation (Table 4), where the model demonstrates consistent performance despite differences in patient demographics and acquisition setups. Low false positive rates, in the range of 0.0038–0.0055, underscore the model’s reliability and clinical practicality, preventing alarm fatigue that often limits deployment of predictive systems. Furthermore, the model incorporates ictal-aware validation aligned with the concepts of Seizure Prediction Horizon (SPH) and Seizure Occurrence Period (SOP), enabling timely alerts up to 5 minutes before seizure onset. Together, these aspects move beyond high accuracy alone, offering a comprehensive framework that enhances generalization, reduces false alarms, and provides clinically actionable early warnings.

While *HybridConvMobileNet* achieves high accuracy and efficiency, it is important to assess its complexity and translational limitations. The architecture integrates CNN feature extractors with MobileNet depthwise separable convolutions, resulting in a compact model of 1.18M parameters and a memory footprint of 4.7 MB after quantization. This makes the framework suitable for real-time execution on embedded devices such as the Typhoon HIL C2000 microcontroller. Nevertheless, further optimization through pruning, quantization-aware training, or hardware-specific accelerators may be necessary for deployment on ultra-low-power wearable devices. Clinical integration also presents risks. Wearable deployment faces constraints of onboard memory, limited battery life, and variability in EEG quality due to electrode shifts, motion artifacts, and noise in ambulatory settings. These challenges can affect long-term robustness and user compliance. Although the proposed model demonstrates low inference latency (0.35 s for a 30 s EEG segment) and minimal memory usage (<6 MB), future research will explore adaptive inference strategies, hardware-aware compression, and hybrid edge–cloud architectures to address these limitations. Tackling these factors is critical for ensuring reliability, safety, and clinical acceptance of wearable seizure prediction systems.

The performance of the *HybridConvMobileNet* model has been thoroughly evaluated against various existing methods. Amrani et al.^25^ combined CNNs with LSTMs, accuracies but at the cost of potential data loss and overfitting due to under-sampling and static configurations. In contrast, the flexible architecture of *HybridConvMobileNet* mitigates these risks, enhancing data integrity and ensuring robust performance, essential for real-time applications. Jana et al.^26^ and Kapoor et al.^28^, while delivering accurate results, are hampered by their high computational demands, limiting their applicability in real-time and low-power environments. In our model, significantly reduces computational requirements while maintaining high performance, ideally suiting it for efficient real-time operations. Another approach by Weiet al.^27^ and Bell et al.^29^, which relies on complex or high-frequency-focused methods, may not consistently perform well across all clinical scenarios. The balanced feature extraction strategy of *HybridConvMobileNet*, however, effectively captures essential EEG details, facilitating comprehensive seizure detection across various conditions. Sonawane et al.^30^ introduced a model that also demands extensive computational resources, which restricts its practical usage. Conversely, *HybridConvMobileNet* achieves comparable or superior accuracy with substantially lower computational needs, reinforcing its suitability for continuous, real-time monitoring. By implementing the *HybridConvMobileNet* model, significant improvements in seizure prediction metrics were observed. On the CHB-MIT dataset, the model reached 99.70% accuracy, 99.31% sensitivity, and a 99.43% F1-score. Similarly, on the Siena dataset, it achieved 99.67% accuracy, 99.08% sensitivity, and a 99.57% F1-score. These results clearly outperform the eight existing methods across both datasets.

A potential misconception is that HybridConvMobileNet relies on a simple concatenation of CNN and MobileNet outputs. In contrast, the ablation results Tables 16–22 demonstrate that each element–multiscale CNN blocks, concatenation, and MobileNet depthwise separable refinement–contributes uniquely. Removal of MobileNet layers or concatenation leads to sharp declines in accuracy and significant increases in FPR. These findings confirm that the fusion mechanism is not trivial but instead represents a systematic integration of complementary modules designed for efficiency and robustness in real-time seizure prediction.

### Real-world application and clinical relevance

The proposed *HybridConvMobileNet* model, with its high accuracy and sensitivity, provides reliable seizure prediction that can support timely therapeutic interventions. Its suitability for integration into clinical workflows and wearable platforms enables continuous EEG monitoring and real-time alerts, thereby enhancing patient safety and healthcare efficiency. Beyond algorithmic validation, it is important to consider the broader seizure prediction landscape. Several commercial systems, such as the NeuroPace RNS^®^ System^45^, the Empatica Embrace^®^ watch^46^, and the Medtronic Percept^TM^ PC neurostimulator^47^, have demonstrated the feasibility of continuous seizure monitoring in clinical practice. These devices represent significant progress in patient management but are primarily limited to seizure detection rather than long-horizon prediction, and their performance often varies substantially across individuals. Clinical deployment further faces challenges such as inter-patient EEG variability, susceptibility to artifacts in emergency recordings, dataset imbalance, and the regulatory requirements for medical device approval. Minimizing false positives remains particularly critical, as unnecessary alarms can cause patient distress or trigger inappropriate interventions. Moreover, most state-of-the-art algorithms are evaluated retrospectively on benchmark datasets, with limited evidence from prospective clinical validation studies, restricting their immediate adoption in real-world healthcare settings. In this context, the *HybridConvMobileNet* contributes a unique advancement by combining >99% accuracy on benchmark datasets with real-time validation on the Typhoon HIL C2000 microcontroller. With sub-second latency and low energy consumption, the model demonstrates a hardware-verified and lightweight design suitable for portable and low-power medical devices. This positions it as a practical step toward bridging the gap between algorithmic innovation and clinically deployable seizure prediction systems.

### Limitations and assumptions

A few limitations and assumptions underlie this study. These include constraints related to dataset size, controlled experimental conditions, and model-specific parameter choices, all of which may influence the generalizability of the results. The evaluation has been conducted on two publicly available benchmark EEG datasets including CHB-MIT and Siena which, although extensively validated and widely used in seizure-prediction research, may not capture the full range of inter-patient and intra-patient variability observed in real-world clinical EEG recordings. Both datasets are limited in terms of patient diversity, recording duration, and electrode placement configurations. Consequently, the performance of the proposed model may vary when applied to larger or heterogeneous clinical datasets encompassing different acquisition protocols, electrode systems, or environmental noise conditions. In terms of data preprocessing, the pre-ictal and inter-ictal segmentation windows have been defined according to established clinical conventions; however, seizure dynamics are inherently patient-specific, and fixed window durations may not fully represent the temporal complexity of pre-seizure transitions. Additionally, the balancing of datasets using SMOTE and sliding-window techniques introduces synthetic variability, which, although effective in addressing class imbalance, may not perfectly emulate physiological patterns of real EEG data. From a modelling perspective, the proposed *HybridConvMobileNet* has been designed for high efficiency and real-time deployment on embedded platforms. While the model exhibits superior accuracy and low false-prediction rates, its performance has been optimized for a specific hardware configuration, the Typhoon HIL C2000 microcontroller. As a result, computational efficiency and latency may vary across different embedded architectures. Furthermore, although MobileNet-based convolutional modules reduce computational complexity, they may slightly limit the model’s ability to capture long-range dependencies in highly non-stationary EEG segments. Future architectural improvements incorporating attention or graph-based representations could enhance feature interpretability and temporal adaptability. Real-time validation has been performed under controlled laboratory conditions, and the system’s robustness under real-world clinical settings where noise, motion artifacts, or incomplete EEG data are common remains to be fully explored. These limitations indicate that while the current findings demonstrate the strong potential of *HybridConvMobileNet* under controlled experimental environments, future research should focus on multi-center clinical validation, dynamic adaptation to patient-specific EEG profiles, and integration of explainable AI mechanisms to further strengthen generalizability, reliability, and clinical trustworthiness.

## Conclusion & future works

This work introduces a unique and efficient technique for seizure prediction using EEG data from both the CHB-MIT and Siena datasets. The proposed *HybridConvMobileNet* model combines the advantages of 1D-CNN and MobileNet architectures to achieve an effective balance of computational efficiency and predictive precision. Leveraging 1D STFT, the model efficiently captures essential frequency-domain features with minimal computational complexity. The model delivers exceptional results, achieving 99.69% accuracy, 99.68% sensitivity, and a 99.11% F1-score on the CHB-MIT dataset, and 99.67% accuracy, 99.08% sensitivity, and a 99.29% F1-score on the Siena dataset. The deployment of *HybridConvMobileNet* on the Typhoon HIL C2000 microcontroller demonstrates its readiness for clinical use, achieving low latency and accuracy needed in real-time settings. Currently, the model adapts to individual EEG patterns to ensure effective performance. Future work will extend energy profiling beyond Typhoon HIL C2000 to platforms such as ARM Cortex-M microcontrollers and FPGA boards, providing a broader validation of power efficiency for real-world wearable and implantable seizure monitoring systems. In parallel, domain-specific loss functions such as FPR-weighted and SPH/SOP-aware formulations together with adaptive optimizers will be explored to enhance clinical reliability, while also developing a generalized, patient-independent version to improve adaptability across diverse patient profiles for broader clinical application. Furthermore, future studies will involve stress testing under clinical conditions with continuous multi-day EEG monitoring, incorporating hospital-level noise, electrode variability, and patient activity to establish robustness and reliability of *HybridConvMobileNet* for real-world deployment and also focus on analyzing canonical EEG frequency bands (delta, theta, alpha, beta, gamma) to enhance interpretability of preictal dynamics and align predictions with established seizure-related biomarkers.

## Data Availability

The datasets used in this study are publicly available. The CHB-MIT Scalp EEG Database can be accessed at https://physionet.org/content/chbmit/1.0.0/, and the Siena Scalp EEG Database is available at https://physionet.org/content/siena-scalp-eeg/1.0.0/.

## Code availability

The *HybridConvMobileNet* implementation used for EEG preprocessing, model training, inference, and real-time deployment has been developed specifically for this study. The complete custom code–including scripts for data preprocessing, 1D STFT feature extraction, model architecture definition, training procedures, evaluation functions, and Typhoon HIL C2000 deployment–is available in a GitHub repository: https://github.com/PriyaranjanBIT/Seamless-Integration-for-Real-Time-Seizure-Prediction-HybridConvMobileNet-on-Typhoon-HIL The code is released under an academic open-source license, allowing unrestricted access for non-commercial research, reproducibility, and further development by the scientific community.
